# Sea anemone and clownfish microbiota diversity and variation during the initial steps of symbiosis

**DOI:** 10.1038/s41598-019-55756-w

**Published:** 2019-12-20

**Authors:** Natacha Roux, Raphaël Lami, Pauline Salis, Kévin Magré, Pascal Romans, Patrick Masanet, David Lecchini, Vincent Laudet

**Affiliations:** 1 Observatoire Océanologique de Banyuls-sur-Mer, UMR CNRS 7232 BIOM; Sorbonne Université Paris; 1, avenue Pierre Fabre, 66650 Banyuls-sur-Mer, France; 2 PSL Research University: EPHE-UPVD-CNRS, USR3278 CRIOBE, BP 1013, 98729 Papetoai, Moorea French Polynesia; 3 Observatoire Océanologique de Banyuls-sur-Mer, USR CNRS 3579 LBBM, Sorbonne Université Paris;1, avenue Pierre Fabre, 66650 Banyuls-sur-Mer, France; 4 FR3724, Observatoire océanologique de Banyuls sur Mer, 66650 Banyuls-sur-Mer, France; 5 Aquarium de Canet-en-Roussillon, 2 Boulevard de la Jetée, 66140 Canet-en-Roussillon, France; 6 Laboratoire d’Excellence “CORAIL”, Moorea, French Polynesia

**Keywords:** Symbiosis, Biodiversity

## Abstract

Clownfishes and sea anemones form an intriguing long-term association, but the mechanism underlying this symbiosis is not well understood. Since clownfishes seem to cover themselves with sea anemone mucus, we investigated the microbiomes of the two partners to search for possible shifts in their compositions. We used a 16S rRNA gene sequencing strategy to study the dynamics of the microbiota during the association between the clownfish *Amphiprion ocellaris* and its host *Heteractis magnifica* under laboratory conditions. The experiment conducted in aquaria revealed that both clownfish and sea anemone mucus had specific signatures compared to artificial sea water. The microbiomes of both species were highly dynamic during the initiation of the symbiosis and for up to seven days after contact. Three families of bacteria (*Haliangiaceae, Pseudoalteromonadacae, Saprospiracae*) were shared between the two organisms after symbiosis. Once the symbiosis had been formed, the clownfishes and sea anemone then shared some communities of their mucus microbiota. This study paves the way for further investigations to determine if similar microbial signatures exist in natural environments, whether such microbial sharing can be beneficial for both organisms, and whether the microbiota is implicated in the mechanisms that protect the clownfish from sea anemone stinging.

## Introduction

Symbiosis, the close and long-term association between two or more organisms of different species, is a fascinating biological phenomenon. Such an association can be beneficial to both partners (mutualism) or to only one of them (commensalism), or it may even be detrimental to one partner (parasitism)^1^. However, many symbiotic relationships are complex and do not necessarily obey these simple distinctions. As all eukaryotic organisms live in symbiosis with complex communities of microorganisms, thus forming holobionts, symbiosis has a key role in shaping ecosystems, food webs and communities. Those associations appear to be increasingly essential for complex organisms^2,3^. However, symbiosis also exists between multicellular organisms. One of the most striking examples is the long-term association between clownfishes and their sea anemones^4^.

Clownfishes, belonging to the Pomacentrids family, are an interesting group for the study of symbiosis. The 28 species of the *Amphiprion* genus and the unique species of *Premnas* can all live in close associations with 3 unrelated families (*Thalassianthidae*, *Actinidae*, *Stichodactilidae*)^5,6^ of sea anemones. Studied since the end of the nineteenth century^7^, this symbiosis is considered to be a mutualistic relationship, as the sea anemones provide protection and nutrients to clownfishes, and clownfishes provide ventilation, nitrogen and carbon to the host and its endosymbiotic zooxanthellae, thus playing an important role in their nutrition^8–10^. Clownfishes also protect their host against predators^4,11,12^.

This symbiosis has always fascinated scientists for two main reasons. First, clownfishes can live safely inside the tentacles of their host, which is known to discharge stinging cells called nematocysts^13^. Second, this mutualistic relationship shows complex species’ specificities, since a few clownfish species live in only one sea anemone species (called specialists, *e.g., A. sebae, P. biaculatus)*, whereas others may have between 2 or even 10 possible hosts (*e.g., A. ocellaris, A. bicinctus, A. perideraion, A. clarkii*)^6,14–16^.

Even though many studies have attempted to better understand the resistance of clownfishes to sea anemone stinging, this question remains unresolved. One reason is probably because the 28 species of clownfishes do not necessarily use the same mechanisms to protect themselves from the various sea anemone species. Two main hypotheses have been formulated to explain the capacity of clownfishes to live safely in their host. The first proposes that clownfishes are protected from sea anemone stinging by their own mucus, which either prevents nematocyst discharge or protects the fish from the consequences of the discharge. Indeed, it has been suggested that *A. sebae* secretes a protective mucus with a similar composition to its host and that *A. ocellaris* lacks *N*-acetylneuraminic acid in its mucus, which is normally detected by sea anemone tentacles to discharge stinging cells^17,18^. The second hypothesis proposes that clownfishes coat themselves with sea anemone mucus, which is therefore used as a chemical camouflage^4,19^. This second hypothesis implies that the fish acclimate to the sea anemones by covering themselves with the sea anemone mucus, thereby becoming gradually invisible to the anemone^13,20^. The acclimation is witnessed by serial changes in clownfish behaviour, when they carefully enter between the tentacles of their host^21^. First, they kiss the tentacles; then, they touch them with their pectoral fins; and finally, they scrub their entire body against the tentacles. This behaviour has been observed in several, but not all, species and seems to differ according to the sea anemone species. For example, *A. clarkii* needs no acclimation when entering *Stichodactyla haddoni*, but it does need to acclimate when entering *Entacmea quadricolor*^13,22,23^. All of these studies suggest that the mucus of the two symbionts is of particular importance for this symbiosis, as it may provide clues about how clownfishes live in sea anemones without being harmed. In addition to its intrinsic chemical composition, the mucus of aquatic living organisms is colonized by numerous microorganisms that can play important roles in host defence and infection prevention^24,25^.

Investigation of the fish skin microbiome is a recent subject of interest for the scientific community. To our knowledge, only two investigations have been reported on the microbiome of Pomacentrid fishes, and only one of them has focused on the potential impact of sea anemone mucus on the microbiome of clownfish mucus^26,27^. Thus, little information is available, and no study has simultaneously recorded both fish skin mucus and sea anemone mucus. Moreover, we found no information about the tropical sea anemone mucus micriobiome^28–32^. Therefore, we studied the microorganisms living in each partner under laboratory conditions before and after the association to (i) describe the microbiota of both the clownfish and sea anemone mucus and (ii) observe whether the compositions of the mucosal microbiota of both the clownfish and sea anemone change during the initial steps of symbiosis.

## Results

### Specificity of the clownfish mucosal microbiome

Before monitoring the changes in the microbiome of the clownfish and the sea anemone mucus during symbiosis, the specificity of the clownfish mucosal microbiome was investigated. The diversity of prokaryotes in the sea water samples from the aquarium was compared with the microbiome compositions of the mucus of five clownfishes. Altogether, the data clustered into 308 different OTUs at a 97% similarity level of 16 S rRNA gene similarity.

**Figure 1:**
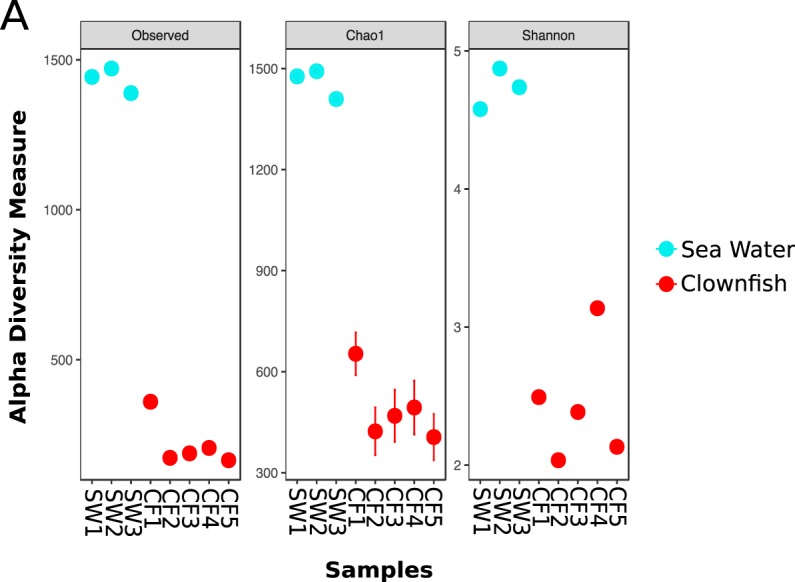
Alpha diversity of the clownfish mucus microbiota (n = 5) in comparison to sea water microbiota (n = 3) with the Chao1 and Shannon indexes respectively showing the richness and phylogenetic diversity of the samples, with sea water being more diverse and richer than the clownfish mucus microbiota.

**Figure 2:**
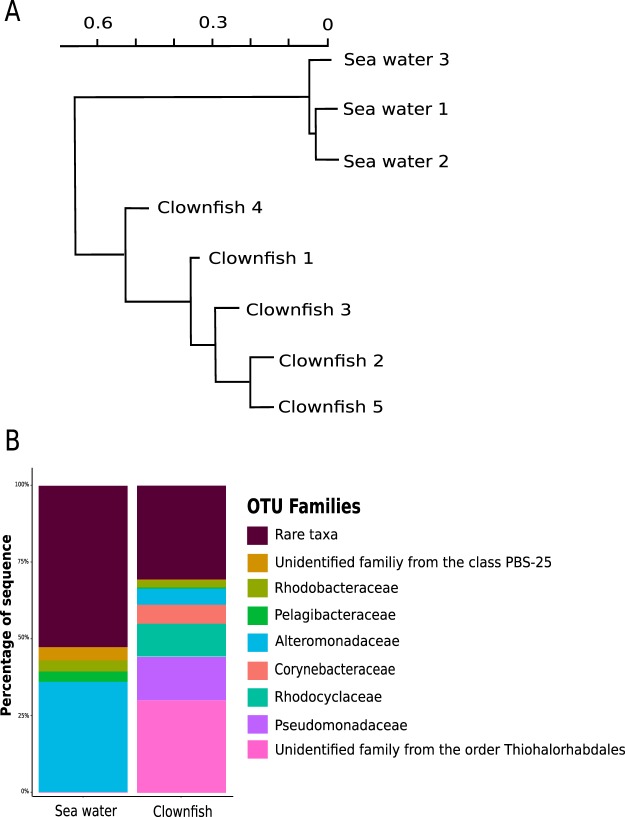
Bacterial diversity and composition of sea water and clownfish mucus samples. (**A**) Dendrogram showing the specificity of the clownfish mucus compared with the sea water microbiomes. (**B**) Taxonomic composition, with the percentages of the most representative families of the microbiomes of clownfish mucus and sea water samples.

The OTU richness and phylogenetic diversity were significantly higher in sea water tank samples compared with clownfish mucus samples, as shown by the diversity index Chao1 and Shannon (Fig. 1, Supplementary. Fig. 1 - sea water: Chao1 = 1459.38 ± 41.68; Shannon = 4.73 ± 0.15; clownfish mucus: Chao1 = 488.83 ± 98.35, Shannon = 2.44 ± 0.43; Kruskal-Wallis test, *p-value* = 0.02535). Similarly, the dendrogram clustering samples based on the OTU composition (Fig. 2A) highlighted a clear distinction between sea water samples and clownfish mucus samples.

This difference was also visible at different taxonomic levels when comparing the relative abundance of the most represented taxa in sea water samples with those detected in clownfish mucus samples (Fig. 2B). Clownfish mucus was mostly composed of bacteria affiliated with the order *Thiohalorhabdales* (mean percentage: 30.01% ± 1.8% of total sequences), as well as with the families *Pseudomonadaceae* (14.05% ± 0.8%), *Rhodocyclaceae* (11.2% ± 0.6%) and *Corynebacteriaceae* (6.1% ± 5.9%). Those communities appeared to be specific to the clownfish mucus. Sea water samples from our aquariums were mostly composed of *Alteromonadaceae* (35.8% ± 2.6%). *Thiohalorhabdales*, *Pseudomonadaceae*, *Corynebacteriaceae* and *Rhodocyclaceae* were also found in sea water, but these families may be considered rare since their abundance was below 1% (0.19% ± 0.0048%, 0.076% ± 0.0087, 0.027% ± 0.0014, 0.053% ± 0.011%, respectively). Even though 209 OTUs (± 70) were shared between clownfish mucus and sea water, 9 OTUs (±39) were strictly specific to clownfish mucus, such as *Chromatiaceae* (0.001% ± 0.002) and *Halociobacillaceae* (0.004% ± 0.004). The high diversity of sea water samples was due to a large abundance of rare OTUs (*i.e*., accounting for less than 1% of the total abundance), which represented approximately 53% (±0.03%) of the total sequences. In contrast, the rare OTUs (<1%) colonizing the clownfish mucus represented a lower proportion of the total OTUs, accounting for 33% (±0.21%) of the total sequences. Overall, these data clearly revealed that clownfish mucus exhibited a specific, less diverse microbiome than sea water.

### Global diversity of the microbiota in clownfish and sea anemone mucus compared with sea water

Before monitoring the change in the microbiome of the clownfish and the sea anemone mucus during symbiosis, the diversity of prokaryotes was compared in the sea water samples from the two experimental aquaria with the microbiome composition of the mucus of the sea anemone and clownfishes (with and without sea anemone). The data were clustered into 308 different OTUs at a 97% similarity level of 16SrRNA gene similarity.

**Figure 3:**
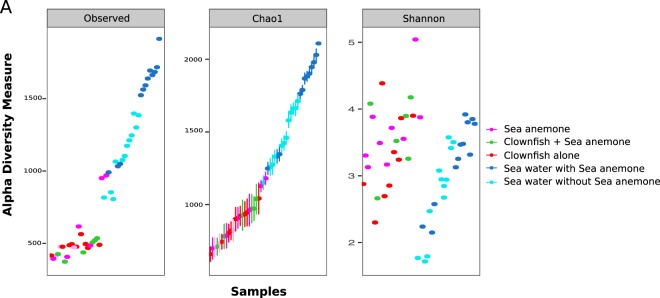
Alpha diversity of the microbiota of clownfish mucus (n = 15), sea anemone mucus (n = 9) and sea water samples (n = 24), with the Chao1 and Shannon indexes respectively showing the richness and phylogenetic diversity of the samples at each sampling time.

The prokaryotic diversity in the aquarium with the sea anemone (tank WSA) was significantly higher compared with the diversity recorded in the aquarium without the sea anemone (tank NSA) (Fig. 3, Supplementary. Fig. 2- maximum Chao1 value for tank WSA = 2109.1355, and for tank NSA = 1712.589; Kruskal-Wallis test, *p-value* = 0.0093; maximum Shannon value for tank WSA = 3.92, and for tank NSA = 3.58, Kruskal-Wallis test, *p-value* =0.46). As expected based on the results presented above, the sea water samples from both tanks (with and without sea anemone) had significantly higher OTU richness and phylogenetic diversity compared with both clownfish and sea anemone mucus (Wilcoxon rank-sum test, *p-value* = 2.4 × 10^−10^ and 1.6×10^−7^, respectively; sea water Chao1 1150.13 - 2109.14, sea water Shannon 1.72–3.92; sea anemone mucus Chao1: 697.36–1180.69, sea anemone mucus Shannon 3.13–5.04). The clownfishes’ mucosal microbiota was less diverse than the sea water samples, as observed previously, with Chao1 values between 659.61 and 1041.59 and Shannon values between 2.30 and 4.39. However, there were no significant differences in OTU diversities between clownfish mucus and sea anemone mucus (Wilcoxon rank-sum test, *p-value* = 0.77). In conclusion, these results are consistent with those presented previously, as sea water has a higher diversity than both clownfish and sea anemone mucus.

### Change in the microbiota of clownfish and sea anemone mucus during the initial steps of symbiosis

**Figure 4:**
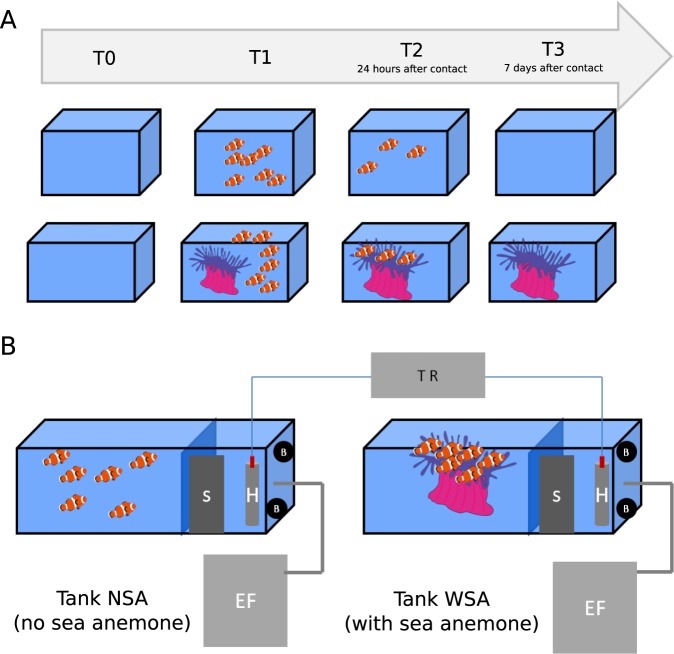
Schematic of the experimental design to monitor the changes in the clownfish and sea anemone mucosal microbiota during the symbiosis, with (**A**) showing the sampling time with T0 sampling before the introduction of organisms, T1 before contact with the sea anemone, T2 24 hours after the first contact and T3 seven days after the first contact. (**B**) The experimental aquaria with skimmers (S), heaters (H) controlled by a thermo regulator (TR), brewing pumps (**B**), and external filters (EF).

To determine how the microbiota composition varied when establishing the sea anemone-clownfish symbiosis, mucus samples of the two organisms and sea water from tanks with no sea anemone (NSA) and with sea anemone (WSA) were sampled and sequenced at different sampling times: before introduction of organisms in the tanks (T0), before contact between clownfishes and sea anemone (T1) and 24 hours (T2) and seven days (T3) after the first contact (Fig. 4A,B). They were clustered into 373 OTUs at a 97% similarity level of 16SrRNA gene similarity. For clarity, the microbiota compositions at these 4 time points are presented separately.

#### T0: Sea water sampling before the introduction of organisms

**Figure 5:**
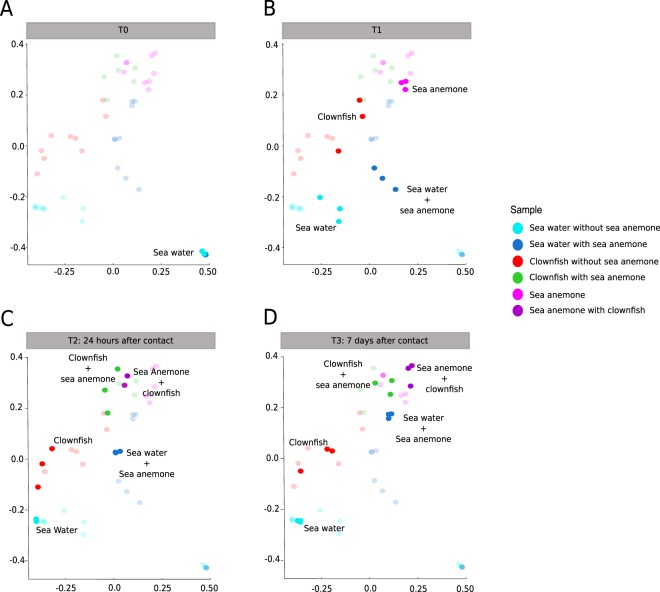
Multidimensional scaling analysis showing the changes in the sea water, sea anemone and clownfish mucus microbiota during the symbiosis (**A**) at T0 prior to the introduction of any organisms in the tanks, (**B)** at T1 before contact with the sea anemone, (**C**) at T2 24 hours after the first contact and (**D**) at T3 seven days after the first contact. Light-coloured points correspond to the sampling time.

**Figure 6:**
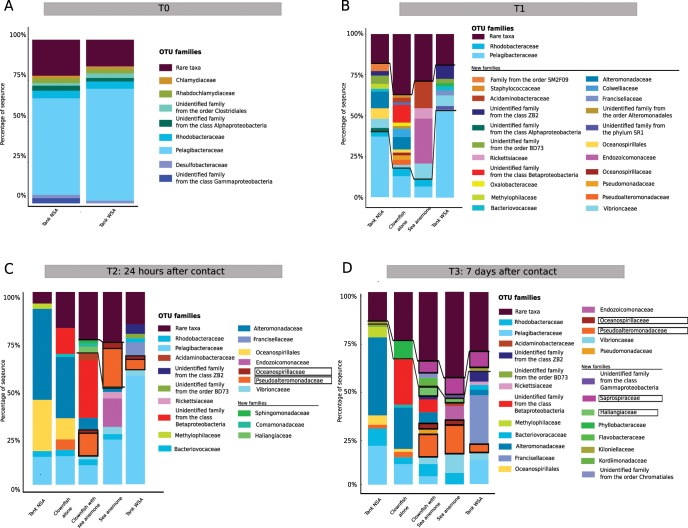
Compositions of sea water and clownfish mucus microbiota (**A**) at T0 before introduction of any organisms in the tanks, (**B**) at T1 before contact with the sea anemone, (**C**) at T2 24 hours after the first contact and (**D**) at T3 seven days after the first contact. Families between the black lines are those that appeared to be very dynamic in the sampled microbiota.

At T0, there were no fishes or anemone in the experimental aquaria (Fig. 4A). The sea water in both tanks presented a very similar microbial composition, sharing a mean of 838.44 OTUs (±15.3) among a total of 965 and 882 OTUs, respectively, in tank NSA and tank WSA. The MDS (multidimensional scaling) confirmed these results, grouping the sea water samples from both tanks together (Fig. 5A). Both tanks were mostly composed of cells affiliated with *Pelagibacteraceae* (SAR11 clade) (tank NSA: 58.9% ± 7%, tank WSA: 68.4% ±3%). Rare taxa (<1% of the total community) represented between 33.6% (tank 1) and 25.9% (tank WSA) of the total sequences (Fig. 6A).

#### T1: Sampling of sea water and organism mucus before contact between sea anemone and clownfishes

At T1, sea anemone had already been introduced in tank WSA, which could explain the observation of a first divergence in the microbial composition in both tanks (Figs. 4A–5B). Even though the majority of taxa in both tanks remained affiliated with *Pelagibacteraceae* (SAR11 clade), this taxa was found to be more abundant in tank WSA than tank NSA (53.6% ± 5%, 37% ± 4%, respectively - Fig. 6B), but this difference was not significant (Kruskal-Wallis test, *p-value* = 0.3173). Many taxa were found to change when comparing the sea water of tank NSA and tank WSA. For example, the following groups were found in tank NSA but not in tank WSA: *Alteromonadaceae* (10% ± 3%), *Oceanospirillales* (6% ± 2%), *Methylophilaceae* (3% ± 1%), *Rhodobacteraceae* (3% ± 1%).

Simultaneously, at T1, the clownfish mucus was mostly composed of *Pelagibacteraceae* taxa, which were also found in the sea water (13% ± 11%) and were already present at T0 (Fig. 6A). However, the proportion of sequences affiliated with this group presented a high interindividual variability between each fish. In accordance with our observations conducted in the first experiment, the clownfish mucus also presented a specific microbial signature compared with the composition of the sea water present in tank NSA. Clownfishes presented on average 134.3 ± 10.5 specific OTUs that were not detected in the sea water. This signature was characterized by the presence of *Betaproteobacteria* (11% ± 11%), *Colwelliaceae* (8% ± 2%), *Pseudoalteromonadaceae* (3% ± 1%), and *Pseudomonadaceae* (3% ± 2%), families that were present at very low levels in sea water (<1% of the total sequences).

The sea anemone mucus alone also demonstrated a distinct microbial signature in comparison to sea water from tank WSA, with 97.6 ± 6.4 OTUs distinct from the sea water. This signature was mostly composed of *Endozoicomonaceae* (27% ± 15%) *Acidaminobacteraceae* (17% ± 5%), *Vibrionacea*e (9% ± 4%), *Pelagibacteraceae* (6.7% ± 1%) and *Rickettsiaceae* (6% ± 5%). Among these, the *Pelagibacteraceae*, *Vibrionaceae* and *Rickettsiaceae* were also well detected in the sea water from tank WSA, and *Acidaminobacteraceae* were also found in clownfishes before they came into contact with sea anemone. In contrast, *Endozoicomonaceae* appeared to be specific to the sea anemone mucus microbial signature, comprising less than 1% of the total sequences in other types of samples.

#### T2: Sampling at 24 hours after contact between sea anemone and clownfishes

At T2, clownfishes and sea anemone had been in contact for 24 hours (Fig. 4A). The MDS analysis revealed that the microbial communities associated with clownfishes alone were clearly distinct from those of clownfishes in contact with sea anemone (Fig. 5C). Indeed, clownfishes from tank NSA (with 463 OTUs ± 40.8 OTUs) shared only 231.6 ± 28 OTUs with clownfishes in contact with anemone in tank WSA (harbouring 444.3 ± 73.8 OTUs). Interestingly, the MDS analysis showed that at T2 (compared with T1), the microbial communities of clownfishes in contact with sea anemone were closer to the communities of the sea anemone mucus. In tank WSA, the clownfishes shared 211 ± 29 OTUs with the sea anemone (444.3 ± 73.8 OTUs in clownfish mucus; 428.6 ± 49.8 OTUs in anemone mucus). This observation was mostly due to a few abundant OTUs presenting quite similar abundances between anemone and clownfish mucus, as the total number of shared OTUs between clownfish and sea anemone mucus did not greatly change (220 ± 10 at T1 and 211 ± 29 at T2).

The divergence of the microbial community in sea water from both tanks was greater 24 hours after the initiation of symbiosis. Indeed, the sea water from tank NSA (without sea anemone) was mostly composed of *Alteromonadaceae* (47.2% ± 0.8%), *Oceanospirillales* (26.5% ± 0.7%) and *Pelagibacteraceae* (14.5% ± 1%). In contrast, the sea water from tank WSA was mostly composed of *Pelagibacteraceae* (56.2% ± 4%), *Francisellaceae* (7.1% ± 1.3%) and *Pseudoalteromonadaceae* (5.3% ± 0.2%) (Fig. 6C).

The microbial community of both clownfishes and sea anemone was still distinct from sea water from both tanks (Fig. 6C). The mucus of clownfishes in both tanks was mainly composed of *Pelagibacteraceae* (clownfishes alone: 14.8% ± 1.1%, clownfishes with sea anemone: 10.1% ± 4.5%) and *Alteromonadaceae* (clownfishes alone: 31.7% ± 2.5%, clownfishes with sea anemone: 6.2% ± 2.8%). The sea anemone mucus was mostly composed of *Pelagibacteraceae* (23.3% ± 1.4%), *Pseudoalteromonadaceae* (20.6% ± 6.1%), *Endozoicomonadaceae* (14.7 ± 8.9%), and *Vibrionaceae* (3.7% ±1.1%). Most of these taxa were clearly abundant in the sea water. In contrast, the mucus of clownfishes in contact with sea anemone also contained three specific families that were not very abundant in sea water: *Haliangaceae* (3.2% ± 5.6%), *Sphingomonadaceae* (1.9% ± 2.5%) and *Comamonadaceae* (1.8% ± 1.9%), but again with high interindividual variability. Interestingly, those three communities were absent in sea anemone mucus and in clownfish mucus without sea anemone not only at T2 but also at T1.

In conclusion, after the initiation of symbiosis between clownfishes and sea anemone, we observed a divergence between clownfishes alone in tank NSA and clownfishes in contact with sea anemone. Both types of clownfishes had a specific microbial signature but distinct associated microbial communities.

#### T3: Sampling at seven days after contact between anemone and clownfishes

At T3, clownfishes and sea anemone had been in contact for seven days (Fig. 4A). As at T2, the MDS analysis showed that the prokaryotic communities associated with clownfishes alone were still different from those of clownfishes in contact with sea anemone (Fig. 5D). Indeed, clownfishes alone in tank NSA (with 512 OTUs ± 45.7 OTUs) shared only 259.2 ± 18.4 OTUs with clownfishes in contact with sea anemone in tank WSA (harbouring 489.6 ± 57 OTUs). The MDS analysis still highlighted that at T3 (compared with T1), the microbial communities of clownfishes in contact with sea anemone were more similar to those in the communities of sea anemone mucus. In tank WSA, clownfishes shared 318.5 ± 41.4 OTUs with anemone (among a total of 489.6 ± 57 OTUs in clownfish mucus and a total of 846 ± 198.5 OTUs in anemone mucus). In contrast to T2, the microbial community of sea anemone mucus was more diverse at T3 (more than 400 news OTUs).

In terms of composition, the sea water microbial communities in both tanks were still composed of different predominant taxa. Indeed, at T3, the sea water from tank NSA (without sea anemone) was mostly composed of *Ateromonadaceae* (40.5% ± 3.6%), *Pelagibacteraceae* (20.2% ± 2.4%) and *Rhodobacteraceae* (9% ± 0.1%), whereas that from tank WSA was mostly composed of *Francisellaceae* (25.5% ± 1%), *Pelagibacteraceae* (12.5% ± 0.5%) and *Saprospiraceae* (8.3% ± 0.1%).

The microbial communities of both clownfishes and sea anemone remained distinct from those of the sea water samples from both tanks (Fig. 6D). Clownfish mucus was specifically composed of *Betaproteobacteria* (23.5% ± 25% for clownfishes without sea anemone, 7.1% ± 5.8% for clownfishes with sea anemone), *Phyllobacteriaceae* (9.9% ± 16% for clownfishes alone), and *Pseudoalteromonadaceae* (2.9% ± 0.3% for clownfishes alone, 11.7% ± 4.8% for clownfishes with sea anemone). Sea anemone mucus was mainly composed of *Saprospiraceae* (8.8% ± 2.1%) and *Endozoicomonadaceae* (7.35% ± 5.4%); however, some communities specific to sea water were also found in the mucus of both living organisms, such as *Alteromonadaceae* (21.4% ± 9% for clownfish alone, 5.9% ± 3.6% for clownfish with sea anemone), *Rhodobacteraceae* (3.5% ± 0.4% for clownfish alone, 6.3% ± 3.9% for clownfish with sea anemone, 5.9% ± 3.2% for sea anemone), *Pelagibacteracea* (10.6% ± 5.2% for clownfish alone, 4.1% ± 2.4% for clownfish with sea anemone) or *Vibrionaceae* (3.8% ± 2.1% for clownfish with sea anemone, 10.2% ± 2.6% for sea anemone). After seven days in contact with each other, a few changes were noted in the bacterial communities of the mucus of both species. Interestingly, three families were shared between clownfishes and sea anemone: *Pseudoalteromonadaceae* (14.9% ± 6.5% for sea anemone, 11.7 ± 4.8% for clownfish), *Saprospiraceae* (8.8% ± 2.1% for sea anemone, 6.2% ± 3.9% for clownfish) and *Haliangaceae* (2.3% ± 1.1% for sea anemone, 4.3% ± 3.9% for clownfish). *Haliangaceae* was specific to clownfish in contact with sea anemone, as this community was also found in clownfish mucus with sea anemone at T2.

**Figure 7:**
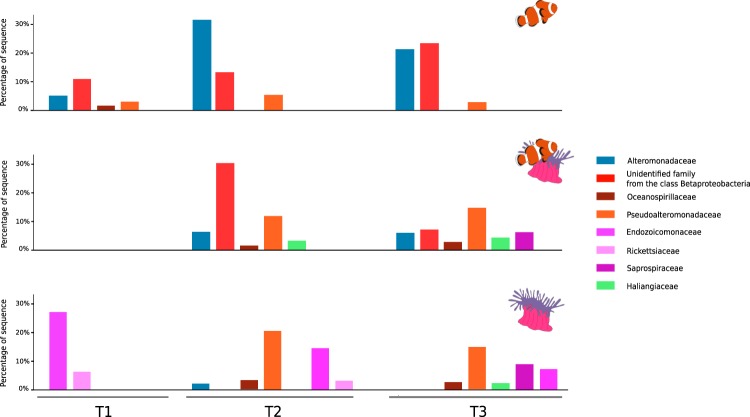
Composition differences between eight relevant microbiota families in clownfish alone, clownfish with sea anemone and sea anemone before symbiosis (T1) and 24 hours (T2) and seven days after symbiosis (T3).

These results indicated that the microbial communities in the mucus of the two symbionts started to mix during symbiosis and were distinct from the sea water from both tanks and from clownfishes without sea anemone (confirmed by the dendrogram shown in Supplementary Fig. 3). These results were also confirmed by a comparison of the change in the most relevant microbial communities between clownfishes without sea anemone, clownfishes with sea anemone and sea anemone mucus (Fig. 7). We observed a very dynamic microbiota over time and noted that the communities shared between sea anemone and clownfishes after symbiosis were not present in clownfish mucus without sea anemone (*Haliangiaceae, Saprospiraceae, Pseudoalteromonadacea*).

## Discussion

High-throughput 16SrRNA sequencing approaches are now widely used to explore the diversity of microbiota and have allowed the characterization of many types of fish microbiomes. However, most of these studies have focused on gut microbiomes (for a review see Lleweyn *et al*.^27^), and works dedicated to skin microbiomes are much less numerous^28,29^. They have also usually concerned the impact of environmental variations on the skin microbiome. For example, a pH drop alters the composition of skin microbiota in the Amazonian fish tambaqui (*Collosoma macropomum*)^33^. Similarly, the salmon microbiome composition is modified during transit from freshwater to sea water^34^. However, to the best of our knowledge, no studies have addressed the question of how the microbiome changes in two marine species that become involved in a symbiotic relationship. The initiation of symbiosis can be considered a habitat transition that may involve environmental changes. Thus, we tested whether, under laboratory conditions, contact between clownfish (*A. ocellaris*) and the sea anemone (*H. magnifica*) has an impact on their mucosal microbiota, changing their compositions, and whether the two partners might share some microbial communities. To test these hypotheses, we set up two experimental aquaria exposing clownfishes to sea anemone in one tank and a control aquarium without sea anemone to confirm that the changes observed in the clownfish microbiome were indeed linked to sea anemone rather than to other experimental parameters.

### Clownfish and sea anemone mucus have specific microbiomes

We first determined that both clownfish and sea anemone mucus have specific microbiome signatures when maintained in a laboratory tank. Clownfish mucus specifically contains *Betaproteobacteria*, regardless of whether the fishes are in contact with sea anemone. Sea anemone mucus contains *Endozoicomonas* and *Rickettsciaceae*, which have also been detected in other cnidarian species, such as the coral *Pocillopora verrucosa*^35^ or the temperate sea anemone *Anemonia viridis*^36^. During the experiment, sea water sample microbiomes were more diverse than those in both clownfish and sea anemone mucus. This phenomenon has been previously observed for not only other fish species, such as *Dicentrarchus labrax* and *Sparus aurata*^37^, but also other cnidarians, such as *Eunicella singularis*, *Eunicella cavolini*, and *Eunicella verrucosa*^38^ (Mediterranean species). Coral reef waters are extremely diverse and contain very diverse and contrasting types of habitats, colonized by very diverse species’ assemblages. Thus, defining a “core bacterial community” associated with a reef’s sea water appears to be very complicated, if such a core community exists. Several reports have clearly pointed out the large variability in coral reef water-associated communities^39–42^. Interestingly, our experimental system permitted the growth and development of bacteria that have been frequently found in field samplings from reefs worldwide, including very diverse types of not only *Proteobacteria*^42^ but also *Pelagibacteraceae, Oxalobacteraceae*, *Alteromonadaceae*^40^ and many others. Thus, our experimental system appears to present many common microbiological traits with a typical sea water reef-associated community, if such a “core community” can be defined.

### The microbiome was highly dynamic and variable during the experiment

We observed that throughout the experiment, the microbiomes of sea water and both clownfish and sea anemone mucus were highly dynamic, with communities disappearing or appearing between sampling times (*i.e., Haliangiaceae, Rickettsiaceae, Alteromonadaceae, Phyllobacteraceae*). Such variability has been observed in aquarium system sea water, showing strong variations despite the stability of the physico-chemical parameters during the survey^43^. For example, bloom events were observed with changes in the predominant communities (*Rhodobacteraceae, Kordiimonas* sp.). According to this study, such fluctuations might be linked to sea water clarity, unmeasured factors or factors beyond the detection limits, or by an input of microorganisms through the filtration system. Thus, one question raised by our results is whether the source of microbial community variation is due to the presence of the sea anemone or to an effect of environmental variables. The observed clustering of both clownfish and sea anemone mucus samples in tank WSA led us to suspect that the sea anemone mucus had a significant effect on the community composition of the clownfish mucus (or reciprocally, the clownfish mucus modified the sea anemone mucus prokaryotic composition). The alternative hypothesis, that other types of environmental variables simultaneously drive the mucus composition of both clownfish and sea anemone, seems less likely. We recorded a large set of environmental data to monitor the quality of sea water in our tanks (temperature, salinity, pH, chlorophyll *a*, pheophytin, nutritive salts). The composition of the sea water remained relatively stable in our aquaria during the experiment and in a range in accordance with that usually observed in marine aquaria. Moreover, the bacterial abundance was stable in both tanks during the course of the experiments and was similar to that commonly found in reef ecosystems (approximately 2×10^6^ cells.ml^−1^ ^44^, Supplementary Table 4D). In addition, we did not observe any development of pathogens, confirming that the quality of our experimental system was good. Nonetheless, our statistical analyses revealed that environmental variables had a significant effect on the prokaryotic community composition (Supplementary Table 4D). We also found that the presence of sea anemone had a significant effect on the community composition of clownfish mucus. It is difficult to disentangle all of these variables because they depend on each other. Changes observed in chlorophyll *a* and pheophytin might be linked to the presence of sea anemone, as cnidarians can enhance their photosynthetic efficiency by increasing chlorophyll *a* abundance in poor light conditions (*e.g., Stylophora pistillata*)^45^. Such changes may also be linked to the increase in organic matter in the experimental aquaria following the introduction of clownfishes and the sea anemone. This increase could also explain changes in nutrient concentrations over time (such as NO_2_^−^ and PO_4_^−^) during our experiments. Elevated values of NO_2_^−^ and NO_3_^−^ at T0 are typical in closed-system aquaria, as they correspond to the nitrogen cycling that leads to stability of the system, as we observed (Supplementary Table 4C). The nutrient concentration in our tanks was then relatively low, stable and consistent with that observed in some coral reefs^46–49^. Despite those variations, the symbiotic relationship between clownfishes and the sea anemone clearly appeared to involve a mix of some of their microbial communities.

Our data also showed the importance of inter-individual microbiota variability between clownfishes (Figs. 2B and 4B); this variability has been previously reported in *Lutjanus campechanus, Cynoscion nebulosus, Sparus aurata*, and *Pangasius hypophthalmus*^37,46,50^. Altogether, these observations suggest that such variability might be an intrinsic feature of fish skin bacterial communities^37^ that can be linked to feeding behaviour, as demonstrated in *Salmo salar*^47^. The skin mucus microbiome composition is dependent on the fish diet. The health status of fishes is also a source of variation in the fish skin microbiome composition^48^. It is possible that this variability arose from the use of different breeding pairs of clownfishes in this study. Even if they were reared and maintained in the same conditions from birth to our experiment, genetic diversity may be involved in inter-individual variability. To our knowledge, no studies have tested the effect of parental diversity on the fish skin microbiome. It would be interesting to compare the inter-individual variability in fish skin microbiomes from the same breeding pairs with those from several breeding pairs.

### Clownfish and sea anemone share some communities after the initiation of symbiosis

As mentioned above, the composition of the microbiota effectively changed during the initial stages of the symbiosis. Interestingly, we observed that seven days after contact, some communities (*e.g., Pseudoalteromonadaceae, Saprospiraceae* and *Haliangiaceae*) were shared between both clownfishes and the sea anemone mucus. To the best of our knowledge, our study is the first to demonstrate the presence of similar microbial communities in the mucus of two organisms living in symbiosis. This result seems to corroborate the hypothesis that clownfishes cover themselves with sea anemone mucus to avoid being stung by the tentacles of their host^13,20^. Interestingly, however, we also found typical clownfish bacterial communities in sea anemone mucus, suggesting that the microbiota of both species had started to mix at seven days after the initiation of symbiosis. Whether sharing their microbiota might play any role in the symbiotic relationship itself remains to be determined. For example, do clownfishes and sea anemone benefit from their mutual microorganisms? The potential role of the microbiota in such relationship is still unknown, but it may be substantial given the prominent metabolic capacities of bacteria. It is possible that bacteria living in the mucus of both symbionts may be involved in the transfer and processing of some nutrients, such as nitrogen and carbon, between the two symbionts^8,9,12^. To tackle this question, a combination of metagenomic, metatranscriptomic and nanostring approaches will enable future studies to target specific functions and gene expression levels within these microbiotas. These approaches could be applied to compare the expression of bacterial functions between anemone-associated fishes and naturally (or experimentally) non-associated fishes. In particular, the production of specific bacterial bioactive metabolites that may play specific roles in symbiosis should be questioned. We are currently testing this possibility experimentally. It would also be very interesting to examine whether such sharing of the microbiota occurs in real situations in the wild. Even if our experimental conditions were as close as possible to those found in reef ecosystems, and our data were in accordance with previous studies on fish and cnidarian mucus microbiomes, we must consider that closed-system aquaria cannot reproduce the variability and dynamic features of the coral reef microbiome. Thus, future studies should include field samplings to confirm our observations. It will also be necessary to sample clownfish eggs before hatching in the field and rear them in circulated system aquaria linked to reef sea water until they reach the juvenile stage. In this way, we might conserve the natural microbiome and characterize how it changes in a natural environment during initiation of the symbiosis between clownfishes and their host. Our study in aquaria was necessary initially to highlight how microbial communities are modified among partners involved in the establishment of a symbiosis under laboratory conditions, thus providing a basis to conduct field experiments.

## Conclusion

Our study allowed the microbial signatures of both symbionts to be compared before and after initiation of the symbiotic relationship in experimental aquaria. Our data revealed that each symbiont harboured a distinct microbial community and that these microbial signatures were significantly modified during the establishment of symbiosis. We also observed that the microbial compositions of both the sea water and the organisms were highly dynamic, showing changes in OTU abundances throughout the experiments (possibly due to experimental conditions). We gained an initial insight about the changes in the microbiota of both clownfishes and sea anemone during initiation of the symbiosis in aquaria. Throughout this process, under our experimental conditions, sea anemone and clownfishes began to share some bacterial taxa in the *Pseudoalteromonadacae, Saprospiracea* and *Haliangiacea* families. It would be of interest to compare these data with *in situ* samplings, although the shared communities may not be the same, since an enclosed sea water aquarium is, of course, very different from the wild environment^30^. *In situ* sampling would enable a comparison of clownfish and sea anemone microbiota when fishes are already settled in their host.

More generally, our results questioned the potential role of the microbiota in symbiotic relationships between eukaryotic organisms, a subject that has been largely neglected until now. Thus, it would be interesting to ascertain whether microbiota play a role in the initiation and/or the maintenance of symbiotic relationships in other cases (*e.g*., carapids/holothurians; shrimps and fishes). There are many interesting cases in marine systems in which the role of the microbiota in symbiotic relationships would be interesting to investigate.

## Material and Methods

### Clownfish and sea anemone maintenance conditions

Clownfishes were obtained from several breeding pairs and were reared in the same tank without sea anemone in our supplier rearing facility. They were thus completely naïve, as they had never been in contact with a sea anemone from the egg to the juvenile stage. Twenty juveniles were transferred to the marine station of Banyuls-sur-Mer (France) and were kept in aquaria for three months before starting the experiments. In this way, the microbiome of the fishes had the time required for stabilization for our experimental conditions^49^. Juveniles of the clownfish *A. ocellaris* and the sea anemone *H. magnifica* were maintained in 2 different aquaria to avoid any contact prior to the experiments. Fishes were kept in a 60-L tank (artificial sea water) at 25 °C with a 12:12-hour light:dark photoperiod. They were fed twice a day with fresh food composed of mussels, shrimp, nori and sardines. The sea anemone *H. magnifica* was chosen because it is the most suitable for aquarium experiments in comparison to the two other natural hosts (*Stichodactyla gigantea* and *S. mertensii*). We have also acquired experience and background data in our teams, as we have already manipulated this species^51^. One sea anemone (imported from Indonesia) was used and maintained in a 100-L tank (closed-system aquarium filled with artificial sea water) at 25 °C in the aquarium of Canet en Roussillon (France) since 2015. We only used one sea anemone to limit our impact on natural populations, by avoiding the retrieval of several organisms from their natural environment. The sea anemone was introduced in the experimental tank one month before starting the sampling so that its microbiome was stabilized^49^. The sea anemone was also fed the same mixture of fishes once a week. Before starting the experiment, the presence of bacterial cells in the mucus of both organisms was confirmed by DAPI staining (data not shown).

### Experimental design

To determine if clownfish mucus has a specific microbiome signature, fishes and the sea water from their tanks were sampled, and DNA was extracted before sequencing. Five fishes were sampled and three water samples of 500 ml, collected in sterile bottles, were pre-filtered (0.8 µm) and re-filtered through 0.2-µm pore size filters before flash freezing for preservation. All samples were conserved at −20 °C until DNA extraction.

To compare the prokaryotic diversity of the mucus of *A. ocellaris* and its host *H. magnifica*, fishes were maintained in closed-system aquaria under two conditions: without sea anemone (Tank NSA) and with sea anemone (Tank SA). Fishes held without sea anemone served as a control to (i) follow the change in the microbiome throughout the experiment, and (ii) to ensure that potential microbiome variations observed in fishes held in tank WSA were effectively due to the presence of the sea anemone. Sea water from the two tanks was sampled one month before the introduction of any organisms to determine the initial prokaryotic diversity of the two experimental aquaria. Then, sea water, fish mucus and sea anemone mucus were sampled at three different times: before the introduction of twelve fishes in the 2 experimental tanks (T1, six fishes per aquarium), at 24 hours (T2) and at seven days (T3) after the fishes had been placed with the sea anemone in tank WSA (Fig. 4A). At each sampling time, three replicates were generated: the mucus of three fishes, three water samples (500 ml each), and three sea anemone mucus samples. All of the sampled fishes were retrieved from the tanks.

Each tank was filled with 100 L of artificial sea water kept at 25 °C. To ensure that the two tanks were at the same temperature, each heater was controlled by the same thermostat (biotherm Pro – Hobby). The same photoperiod was applied (12:12 hours light:dark). Each tank was equipped with two brewing pumps, a skimmer (Tunze Comline Doc Skimmer) and an external filter (Eheim Pro 4) in closed-system aquaria. All of the equipment was separated from the rest of the tank with a grid to avoid any injuries to the sea anemone and fishes (Fig. 4B). To ensure that any changes in microbiome composition of fishes and/or sea anemone were linked to the symbiosis and not to changes in our experimental conditions, different physico-chemical parameters were monitored throughout the experiment: bacterial abundance (cytometry), nutrients, chlorophyll concentrations (following previously published protocols^52–54^), temperature, salinity and pH. No water changes were conducted during the experiment, and no other compounds, such as antibiotics or antibacterial agents, were added to the water to control the bacterial population, since the bacterial population densities were stable throughout the experiment. The organisms were fed once a day with the same quantity of food to avoid any leftovers and to increase the nutrient concentrations.

### DNA extraction

Fishes were anaesthetized individually an MS222 solution at 100 mg/l, placed in a sterile petri-dish (changed after each individual), and measured, and their mucus was sampled with a sterile swab from both flanks of the fish. Precautions were taken to avoid touching the gills with the swab due to the differences in gill and skin microbiomes^48^. The swab was scrubbed three times on each flank up to the tail. A swab-based protocol was chosen as the sampling method, as it causes no harm to the fishes, removes few epidermal cells, and limits superficial abrasion^55^. The cotton was then removed from the plastic rod with a sterile scalpel blade introduced into a 2-ml tube and flash frozen in liquid nitrogen. To sample the sea anemone mucus, the organism was removed from the water, and the mucus was quickly collected from the tentacles with a sterile swab. Three samplings were performed with three different swabs, taking care to sample the same tentacles. The cotton was then removed from the swabs as described for the fish samples. Sea water was collected as described above. All samples were kept at −20 °C before DNA extraction.

To extract DNA, each sample was crushed in liquid nitrogen with a sterile mortar and pestle. The Qiagen allPrep DNA/RNA mini kit was used to extract total DNA following the manufacturer’s recommendations. DNA concentrations were then measured with a NanoDrop (Agilent Plus). Samples were kept at −20 °C before sequencing.

### 16SrRNA gene sequencing and analysis

The 16S rRNA sequencing process was performed commercially at Mr DNA (http://www.mrdnalab.com, Shallowater, TX, USA) by applying routinely used MiSeq-based pipelines. Briefly, the diversity and composition of prokaryotic communities were analysed by amplifying the V4 hypervariable region of 16SrRNA genes, based on PCR amplification using the universal primer set 515F/806R^56^. DNA samples were amplified with a HotStarTaq Plus Master Mix kit (Qiagen, Hilden, Germany) under the following conditions: 94 °C for 3 min, followed by 28 cycles of 94 °C for 30 sec, 53 °C for 40 sec and 72 °C for 1 min, followed by a final elongation step at 72 °C for 5 min. After amplification, PCR products were checked in 2% agarose. Multiple samples were pooled together in equal proportions. Pooled samples were purified using calibrated Ampure XP magnetic beads (Beckman Coulter, Inc., Pasadena, USA). Then, the pooled and purified PCR products were used to prepare an Illumina DNA library. Paired-end sequencing (2 × 300 bp) was performed on the Illumina MiSeq sequencing platform (Illumina, San Diego, USA).

The raw MiSeq paired-end reads from 16SrRNA gene fragments were separately assembled and reoriented using the Mr DNA pipeline. Then, all of the reads were quality filtered with a maximum expected error threshold of 1^57^ and a minimum length of 300 bp, dereplicated and sorted by abundance after singleton removal^58^. Sequence analysis was then performed using QIIME (Quantitative Insights Into Microbial Ecology, http://www.qiime.org)^59^, except for the chimaera removal step and the OTU (operational taxonomic unit grouped by DNA sequence similarity) clustering step, which were performed using UPARSE^58^. OTUs were clustered at >97% identity to OTU centroid sequences (representative sequences that were selected based on their rank of read abundance after dereplication). Complementary data analyses were also performed using the package phyloseq.^60^, and a multidimensional scaling (MDS) was performed to visualize the level of similarity between each sample throughout the experiment.

### Statistical analysis of environmental variables effects

A canonical correspondence analysis (CCA) based on the vegan package^61^ was used to investigate the variations in OTU data under the constraints of our set of environmental variables. Significant variables (*i.e*., variables that significantly explained changes in community composition) in our data set were chosen using a forward-selection procedure and 999 permutations (envfit function in the vegan package)^61,52–54^. In all of these analyses, we assumed a unimodal response of species to environmental variations, meaning that the species had only one clear peak of abundance according to environmental variations.

### Ethics approval

The animals were maintained in our aquarium facility prior to the experiments. No animals were killed for this study; fishes were only anaesthetized, and they were all reintroduced in our aquarium facility once they had recovered from anaesthesia in a quarantined aquarium. The experiments were thus conducted in accordance with the relevant guidelines and regulations. We obtained approval for these experiments from the C2EA - 36 Ethics Committee for Animal Experiment Languedoc-Roussillon (CEEA-LR), number A6601601. The approval number for the premises for animal testing, issued by the Regional Directorate of Food, Agriculture and Forestry of Occitania and the Departmental Directorate of Protection of Populations of the Pyrenees Orientales, is A6601601. The experimental protocols were based on the regulations in force in France (Articles R214-87 to R214-137 of the Rural Code), updated by Decree 2013-118 and by five decrees dated February 1, 2013, and published on February 7, 2013, pursuant to Directive 2010/63/EU. This regulation is under the responsibility of the Ministry of Agriculture.

## Supplementary information

Supplementary Figures and Table

## Data Availability

The datasets generated and analysed during the current study were submitted to the NCBI SRA database under the following accession number: PRJNA499037.
