# Supplementary material for: Sea anemone and clownfish microbiota diversity and variation during the initial steps of symbiosis
Source: Sci Rep. 2019 Dec 20;9:19491. doi: 10.1038/s41598-019-55756-w (PMC6925283; doi:10.1038/s41598-019-55756-w)
Supplement: Supplementary file 1 — Supplementary Figures and Table [file 41598_2019_55756_MOESM1_ESM.pdf]

Sea anemone and clownfish microbiota diversity and variation during the initial steps of the symbiosis

Natacha Roux[1,2*], Raphaël Lami[3*], Pauline Salis[1], Kévin Magré[1], Pascal Romans[4], Patrick Masanet[5], David Lecchini[2,6] and Vincent Laudet[1$].

1: Observatoire Océanologique de Banyuls-sur-Mer, UMR CNRS 7232 BIOM; Sorbonne Université Paris; 1, avenue Pierre Fabre, 66650 Banyuls-sur-Mer, France

2: PSL Research University: EPHE-UPVD-CNRS, USR3278 CRIOBE, BP 1013, 98729 Papetoai, Moorea, French Polynesia

3: Observatoire Océanologique de Banyuls-sur-Mer, USR CNRS 3579 LBBM, Sorbonne Université Paris;1, avenue Pierre Fabre, 66650 Banyuls-sur-Mer, France

4: FR3724, Observatoire océanologique de Banyuls sur Mer, 66650 Banyuls-sur-Mer, France

5 : Aquarium de Canet-en-Roussillon, 2 Boulevard de la Jetée, 66140 Canet-en-Roussillon, France

6 : Laboratoire d'Excellence "CORAIL", Moorea, French Polynesia.

*Equal first author

$ Corresponding author: Vincent.Laudet@obs-banyuls.fr

Key words: Sea anemone, Clownfish, Symbiosis, Microbiota.

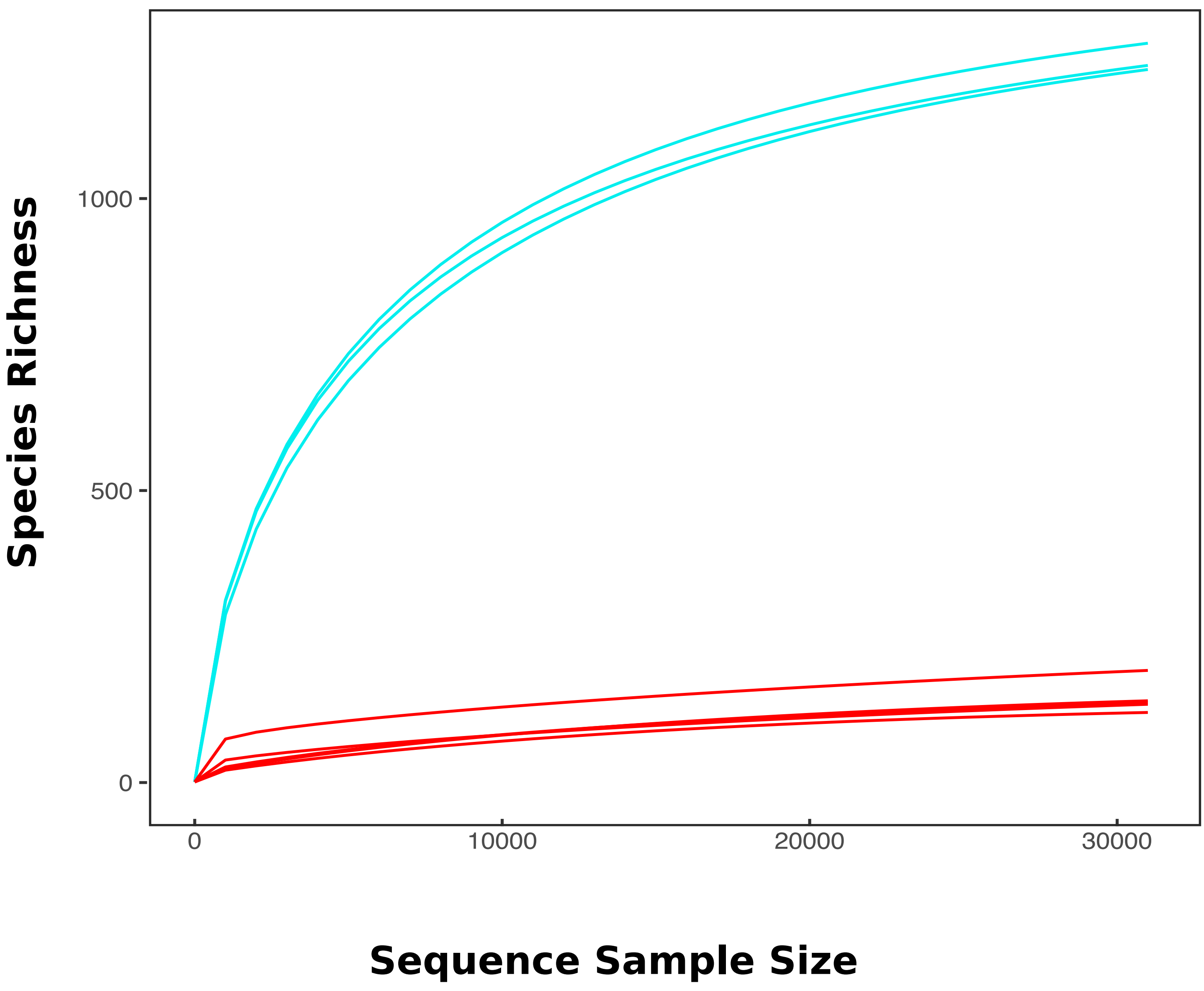

Supplementary Figure 1: Rarefaction curves of clownfish mucus microbiota (n=5) compared to sea water microbiota (n=3).

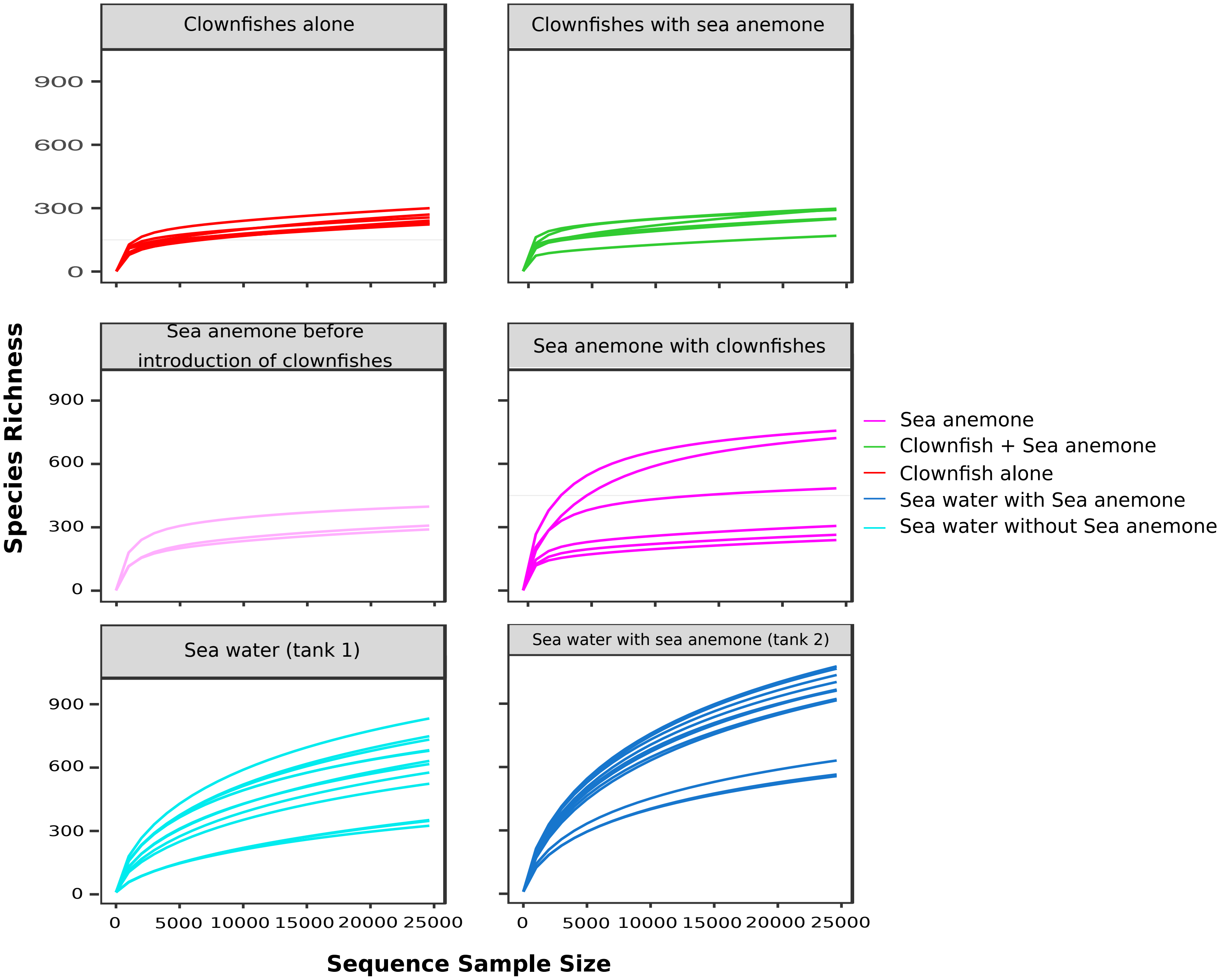

Supplementary Figure 2: Rarefaction curves of the microbiota of clownfish mucus (n=15), sea anemone mucus (n=9) and sea water samples (n=24)

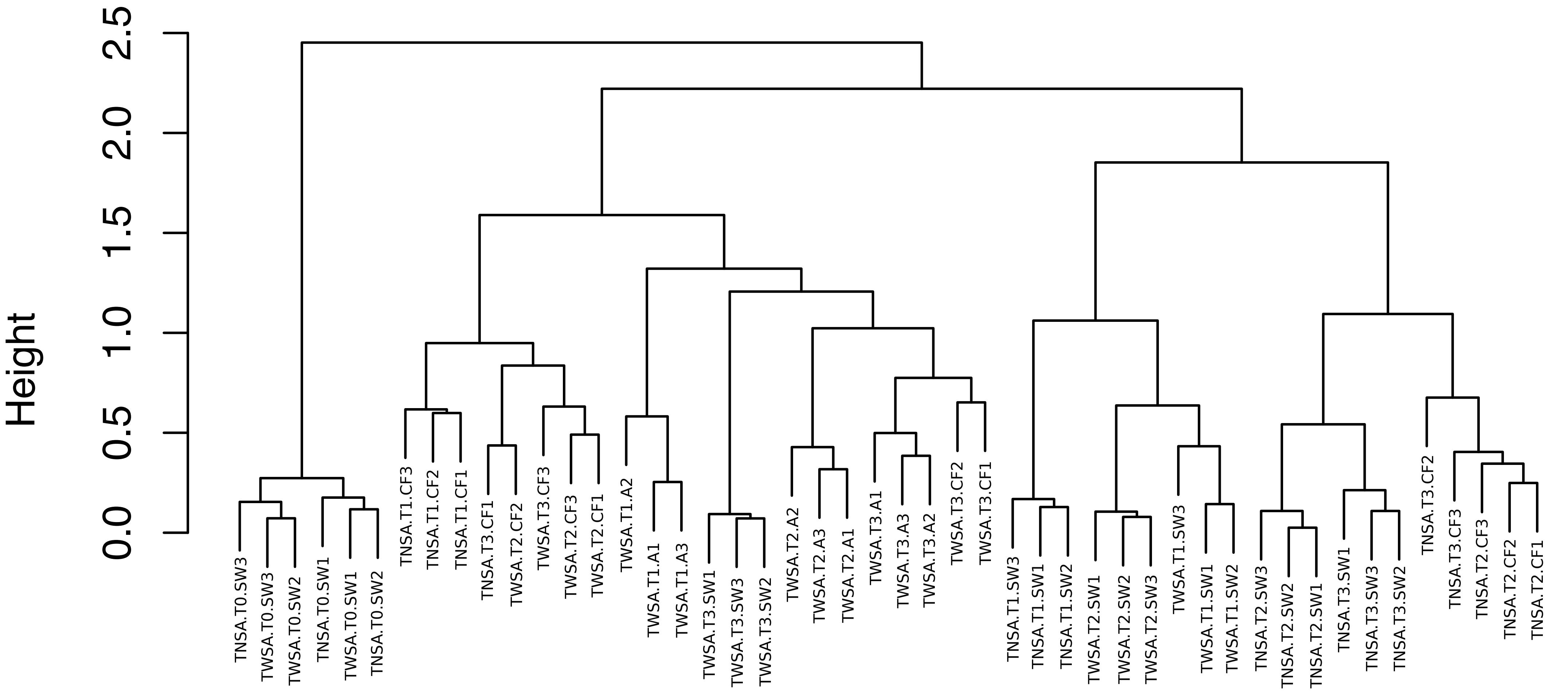

Supplementary Figure 3: Dendrogram showing the specificity of the clownfish and sea anemone mucus microbiota compared to sea water and clownfish alone. TWSA: Tank With Sea Anemone, TNSA: Tank without Sea Anemone, T0: Time 0 (before introduction of sea anemone and clownfishes), T1: Time 1 (after introduction of sea anemone and clownfishes),T2: Time 2 (24 hours aftercontact between sea anemone and clownfishes, T3: Time 3 (7 days after contact between sea anemone and clownfishes), SW: Sea water, CF: Clownfish

A

Time	Temperature		Salinity		pH	
	Tank NSA *(0.016)*	Tank WSA *(0.0001)*	Tank NSA *(0.011)*	Tank WSA *(0.001)*	Tank NSA *(0.001)*	Tank WSA *(0.05)*
T0	24.7	24.6	33.1	33	8.33	8.29
T1	24.6	25.3	32.9	32.8	8.36	8.29
T2	25.6	25.2	32.9	32.8	8.27	8.12
T3	24.6	25.2	32.8	33.2	8.32	8.12

B

Time	Chlorophyll *a*		Pheophitine	
	Tank NSA *(0.305)*	Tank WSA *(0.002)*	Tank NSA *(0.413)*	Tank WSA *(0.231)*
T0	0.024	0.009	0.004	0.003
T1	0.303	2.229	0.049	0.388
T2	0.335	2.229	0.048	1.734
T3	1.575	0.614	0.834	0.219

C

Time	NO2		$NO3^-$		PO4		SiO4	
	Tank NSA *(0.136)*	Tank WSA *(0.001)*	Tank NSA *(0.007)*	Tank WSA *(0.002)*	Tank NSA *(0.001)*	Tank WSA *(0.001)*	Tank NSA *(0.011)*	Tank WSA *(0.005)*
T0	0.117	0.092	30.503	31.548	0.01	0.01075	6.707	6.512
T1	0.026	0.079	0.03525	0.3485	0.01275	0.039	0.387	0.472
T2	0.0585	0.142	0.309	0.7205	0.03	0.04	0.407	0.262
T3	0.1655	0.3555	0.142	4.632	0.029	0.0665	0.052	1.017

D

Time	Bacterial abundance (C/ml)	
	Tank NSA *(0.013)*	Tank WSA *(0.012)*
T0	$9.51x10^5$	$1.20x10^6$
T1	$1.08x10^6$	$1.73x10^6$
T2	$5.34x10^6$	$1.99x10^6$
T3	$2.14x10^6$	$2.18x10^6$

Supplementary Table 1: Physico-chemical parameters controlled during the sampling of sea water, clownfish and sea anemone mucus before, 24 hours and seven days after symbiosis in each experimental aquaria with in A) temperature, salinity and pH, in B) chlorophyll *a* and Pheophitine, in C) nutrients salts and in D) bacterial abundances. Values between brackets correspond to the *p-value*.
